# Supplementary figures and images for: Variable microbiomes between mosquito lines are maintained across different environments
Source: PLoS Negl Trop Dis. 2023 Sep 25;17(9):e0011306. doi: 10.1371/journal.pntd.0011306 (PMC10553814; doi:10.1371/journal.pntd.0011306)

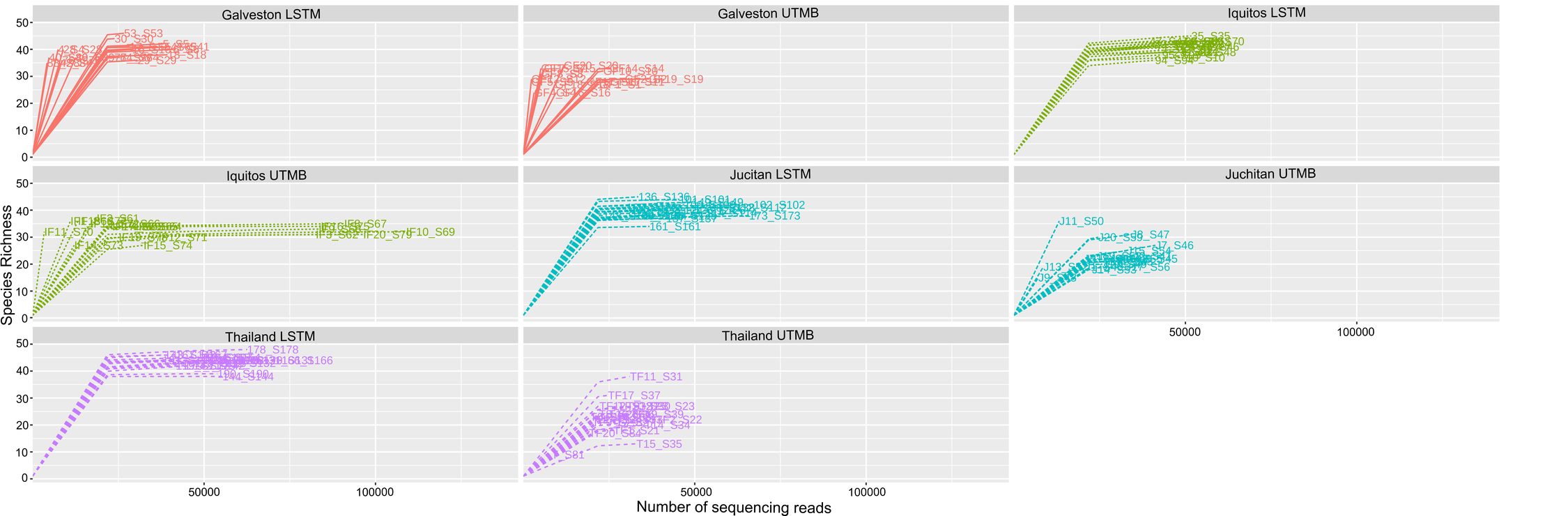

Supplement: S1 Fig — The number of species is shown on the Y axis, and the number of sequencing reads is shown on the X axis. (TIF) [file pntd.0011306.s004.tif]

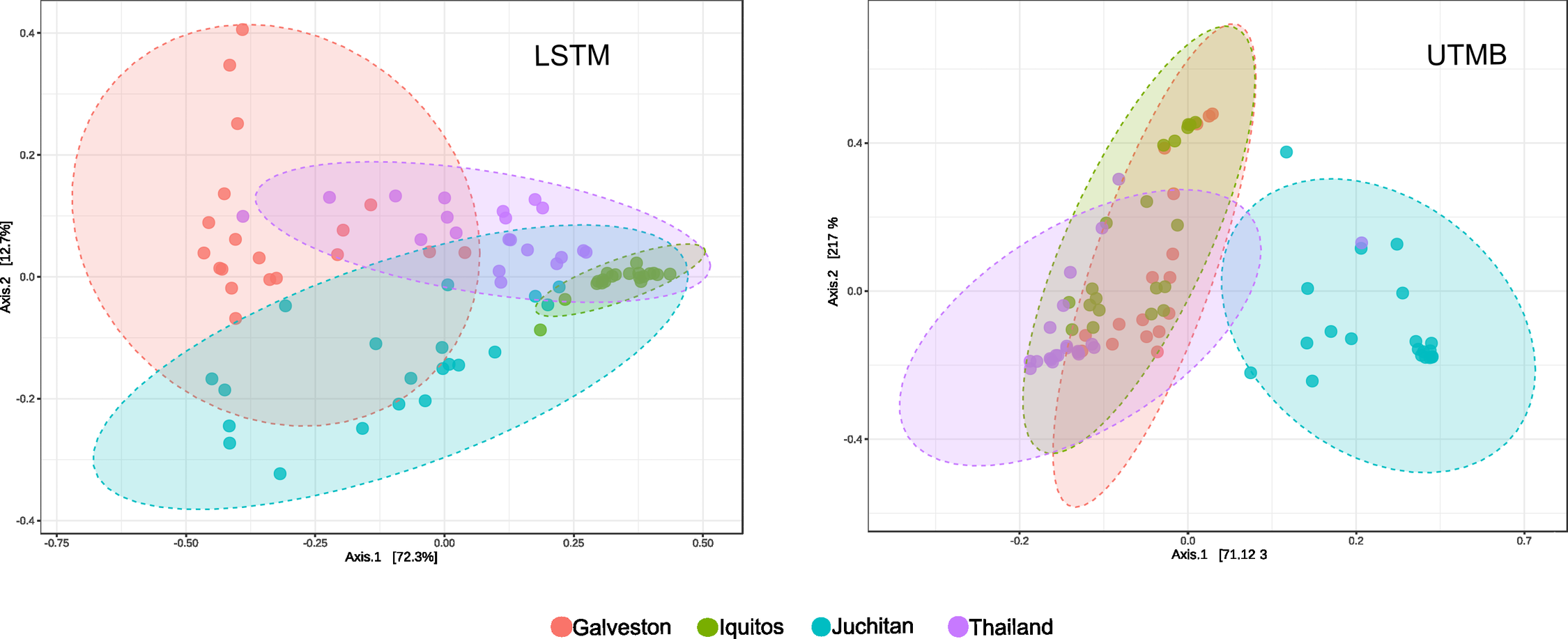

Supplement: S2 Fig — The dissimilarities between the 4 different lines of Ae. aegypti at each insectary were analyzed by principal component analysis of Bray-Curtis dissimilarity index. (TIF) [file pntd.0011306.s005.tif]

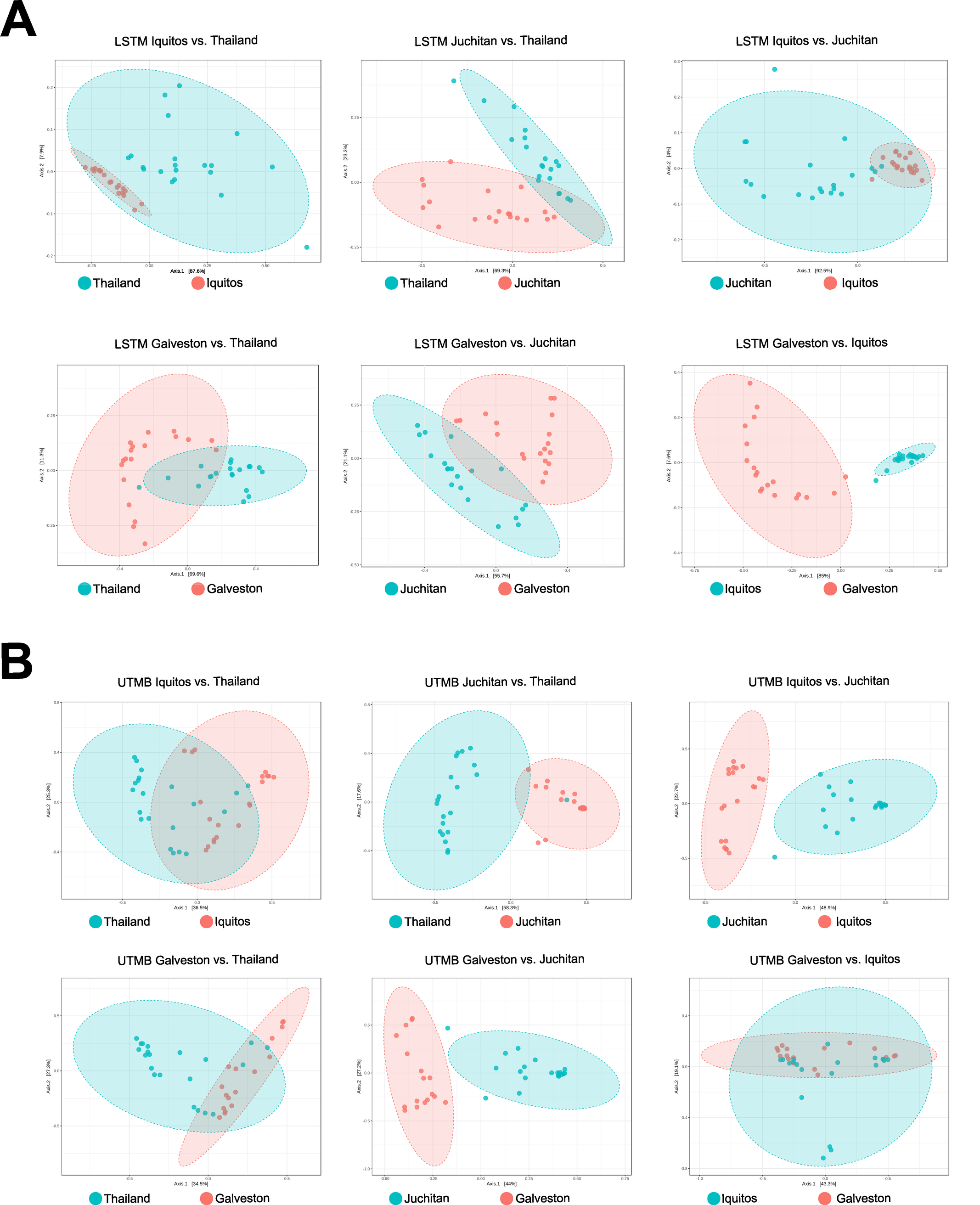

Supplement: S3 Fig — The dissimilarities between the pairs of lines of Ae. aegypti at each insectary were analyzed by principal component analysis of Bray-Curtis dissimilarity index. PERMANOVA analysis, LSTM: Iquitos vs Thailand p-value = 0.001, Juchitan vs Thailand p-value = 0.001, Iquitos vs Thailand p-value = 0.001, Galveston vs Thailand p-value = 0.001, Galveston vs Juchitan p-value = 0.001, Galveston vs Iquitos p-value = 0.001. UTMB: Iquitos vs Thailand p-value = 0.001, Juchitan vs Thailand p-value = 0.001, Iquitos vs Thailand p-value = 0.001, Galveston vs Thailand p-value = 0.001, Galveston vs Juchitan p-value = 0.001, Galveston vs Iquitos p-value = 0.001.) (TIF) [file pntd.0011306.s006.tif]

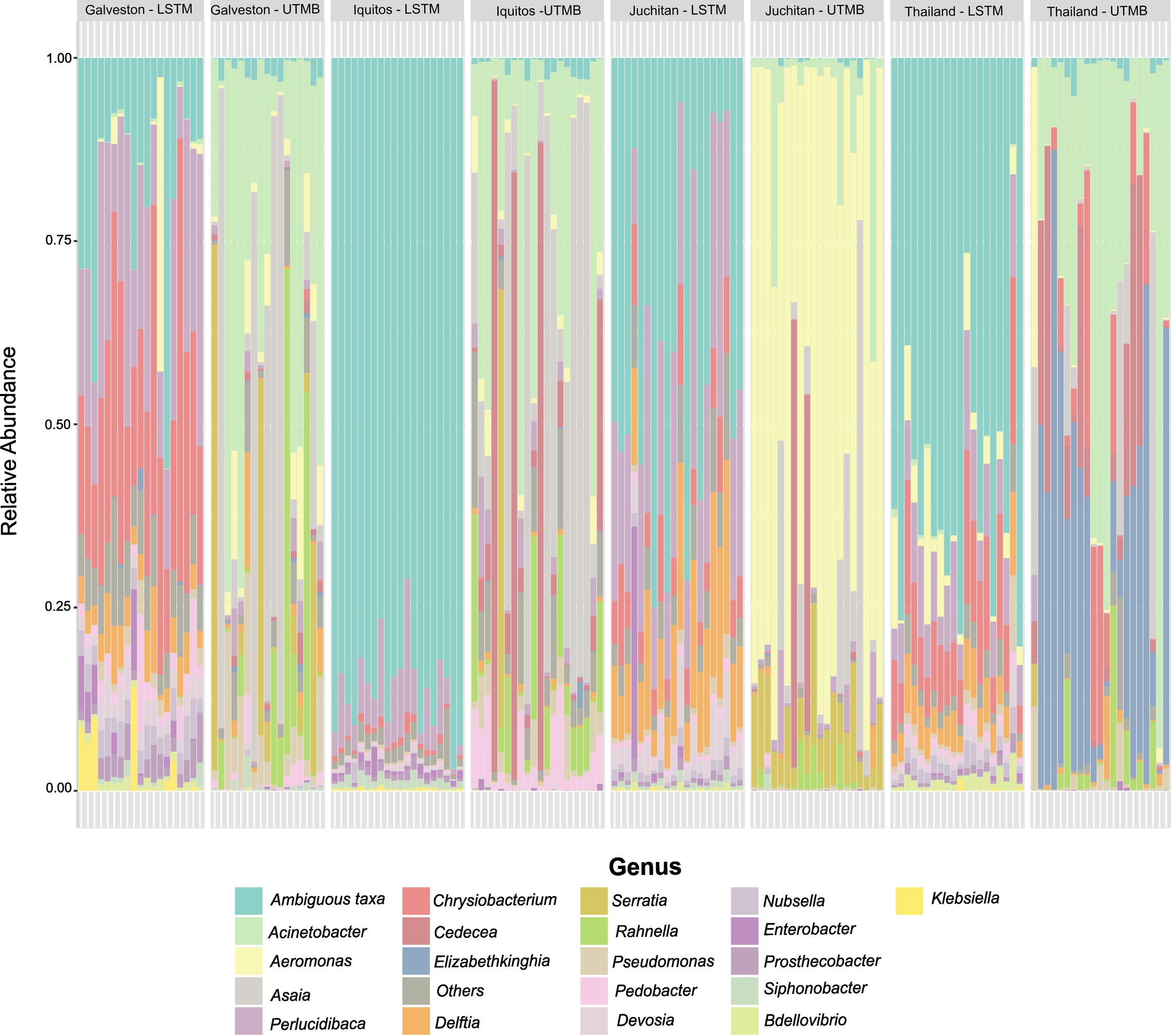

Supplement: S4 Fig — Bacterial genera were assigned to OTUs clustered with a 97% sequence identity cutoff and taxonomically classified with the SILVA database. (TIF) [file pntd.0011306.s007.tif]

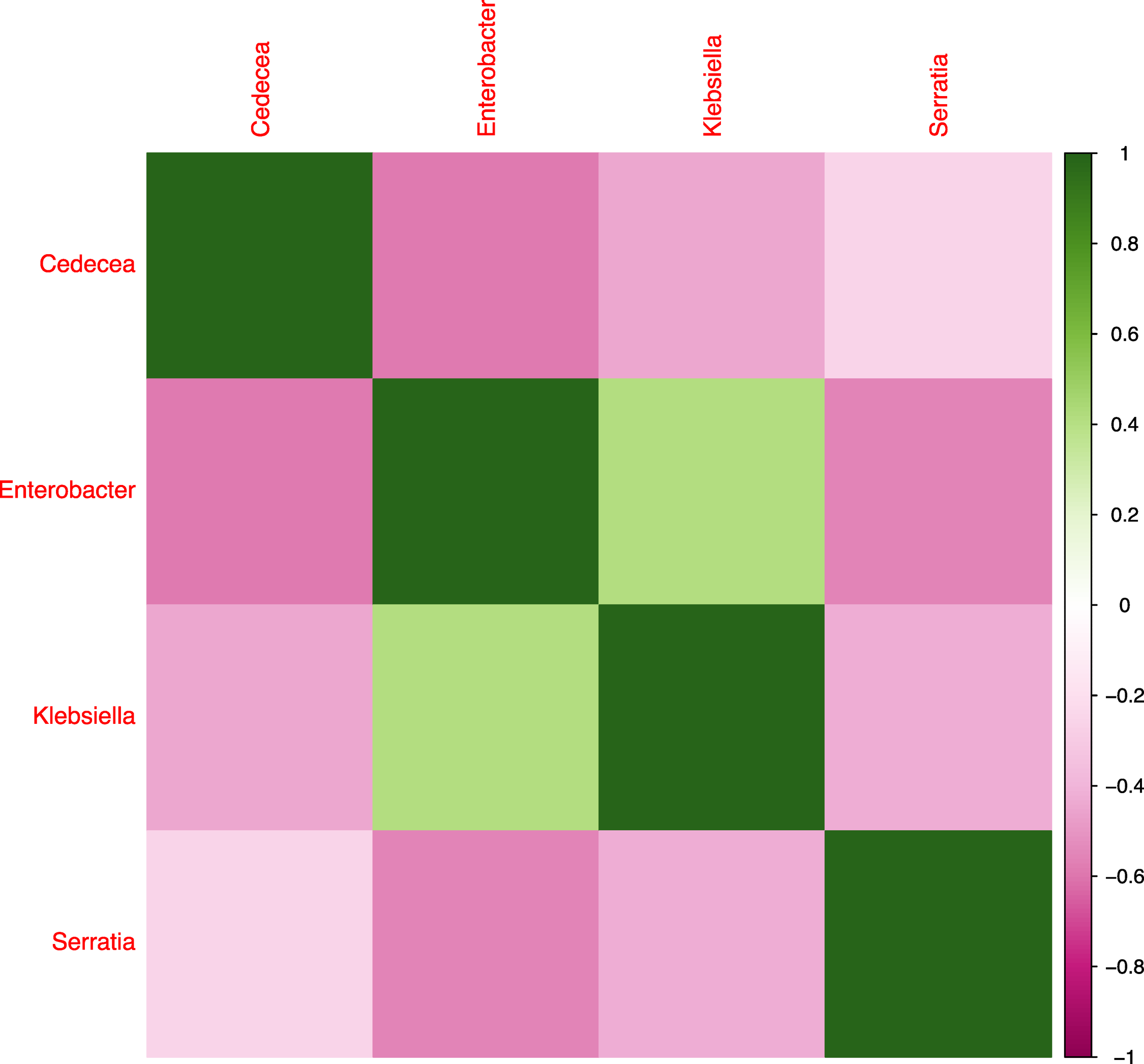

Supplement: S5 Fig — Saturated greens indicate strong co-occurrence between the bacteria taxa, while saturated purple indicates strong exclusion, and whites indicate no positive or negative correlation. We show that Klebsiella co-occurs with Enterobacter taxa, whilst Serratia and Cedecea taxa appear to exclude other bacterial taxa. (TIF) [file pntd.0011306.s008.tif]
